# Rapid microbial identification and colistin resistance detection via MALDI-TOF MS using a novel on-target extraction of membrane lipids

**DOI:** 10.1038/s41598-020-78401-3

**Published:** 2020-12-09

**Authors:** Matthew Sorensen, Courtney E. Chandler, Francesca M. Gardner, Salma Ramadan, Prasanna D. Khot, Lisa M. Leung, Christine E. Farrance, David R. Goodlett, Robert K. Ernst, Erik Nilsson

**Affiliations:** 1 Pataigin, LLC, Seattle, WA USA; 2 University of Maryland, Baltimore, Baltimore, MD 21201 USA; 3 Charles River Laboratories, Inc., Newark, DE 19711 USA; 4 Maryland Department of Health and Mental Hygiene, Baltimore, MD 21205 USA; 5 International Centre for Cancer Vaccine Science, University of Gdansk, Gdańsk, Poland; 6 Present Address: Johns Hopkins University, Baltimore, MD 21205 USA; 7 Present Address: U.S. Food and Drug Administration, Silver Spring, MD 20993 USA

**Keywords:** Infectious-disease diagnostics, Membrane lipids, Mass spectrometry, Microbiology techniques, Biochemical assays

## Abstract

Rapid infection diagnosis is critical to improving patient treatment and outcome. Recent studies have shown microbial lipids to be sensitive and selective biomarkers for identifying bacterial and fungal species and antimicrobial resistance. Practical procedures for microbial lipid biomarker analysis will therefore improve patient outcomes and antimicrobial stewardship. However, current lipid extraction methods require significant hands-on time and are thus not suited for direct adoption as a clinical assay for microbial identification. Here, we have developed a method for lipid extraction directly on the surface of stainless-steel matrix-assisted laser desorption/ionization time-of-flight mass spectrometry (MALDI-TOF MS) plates, termed fast lipid analysis technique or FLAT, which facilitates the identification of bacterial and fungal species using a sub-60-minute workflow. Additionally, our method detects lipid A modifications in Gram-negative bacteria that are associated with antimicrobial resistance, including to colistin.

## Introduction

Rapid administration of appropriate antimicrobial therapy significantly improves patient survival^1,2^, making rapid microbial diagnostics a critical need. Matrix-assisted laser desorption/ionization time-of-flight mass spectrometry (MALDI-TOF MS or simply MALDI) of proteins in the 2000 to 20,000 *m/z* range is the clinical standard for microbial identification (ID)^3^. MALDI protein ID offers a simple workflow, low cost-per-sample, and rapid time to result using colonies from solid growth medium. Current MALDI ID systems include MALDI Biotyper (Bruker Daltonics, Billerica, MA) and VITEK MS (bioMérieux, France). These mature platforms still have significant limitations. Identification at the species level can be unreliable for some common clinical species. For example, *Shigella* cannot be reliably distinguished from *Escherichia coli*. Finally, while researchers report MALDI-based approaches to antimicrobial resistance, MALDI-based clinical tests are not available or limited for antimicrobial resistance^4–10^. Consequently, antibiotic susceptibility testing typically requires sample culture in appropriate growth mediums to obtain pure cultures, followed by parallel subculture in serially-diluted antibiotics, potentially requiring days to weeks depending on the growth rate of the microbe. As multi-drug resistant bacteria and fungi continue to emerge**,** rapid determination of antimicrobial resistance is increasingly critical to antimicrobial stewardship^11–14^. Current early resistance tests such as PCR-based assays have made important contributions, but the expense of these tests limits them to specific applications^13,15^.

Leung et al*.* reported a novel MALDI-TOF MS method (BACLIB) using microorganism-specific lipids in the 1000–2400 *m/z* range as a species-specific “chemical barcode” primarily comprised of lipid A in Gram-negative bacteria and of cardiolipins and lipoteichoic acid in Gram-positive bacteria^16^. Similar to MALDI protein ID, they reported a lipid reference library with entries for over 50 species, including the clinically-relevant ESKAPE pathogens (*Enterococcus faecium, Staphylococcus aureus, Klebsiella pneumoniae, Acinetobacter baumannii, Pseudomonas aeruginosa*, and *Enterobacter* spp.) and intrinsically colistin-resistant bacteria, such as *Morganella morganii* and *Serratia marcescens*^16^.

The BACLIB approach exploits the diverse yet consistent membrane lipids present in bacterial and fungal species. Gram-negative bacterial membranes contain lipid A, the lipid anchor of lipopolysaccharide (LPS), comprising a β-1′,6-linked diglucosamine backbone, a species-specific pattern of four to seven fatty acyl chains, and one or two terminal phosphate groups. Lipids extracted from Gram-positive bacterial membranes include cardiolipins and lipoteichoic acid (LTA), which also have species-specific patterns^16^. Fungal membranes are composed of phospholipids, glycosphingolipids, and sterols^17^.

Furthermore, antimicrobial resistance can be mediated via modification of membrane lipids. For example, addition of phosphoethanolamine, l-amino-4-arabinose (Ara4N), or galactosamine to the above-mentioned lipid A terminal phosphate groups reduces membrane permeability to polymyxins, such as colistin (polymyxin E)^18,19^. Membrane lipid modifications conferring antimicrobial resistance have been characterized by MALDI^18–29^. Subsequent to reporting the BACLIB method, Leung et al*.* showed the ability to determine colistin resistance MALDI patterns in the ESKAPE pathogens (*Klebsiella pneumoniae, Acinetobacter baumannii*) and those driven by the *mcr-1* gene, a phosphoethanolamine transferase^20,21,26,28,29^. Thus, MALDI is an attractive platform for a rapid resistance assay and the approach of Leung et al*.* yields a single test combining multiplex microbial ID and resistance assays. However, the approach in Leung et al*.* involves centrifugation and labor-intensive washing steps and requires overnight lyophilization—impractical for a clinical test. Liang et al*.* describe a 1-h sodium acetate-based lipid extraction^28^, but the protocol also includes centrifugation and ethanol washing steps that are problematic for a clinical technique. Furniss et al*.* recently described a rapid lipid extraction using acetic acid^8^, but as with Liang et al., centrifugation and labor-intensive handling steps are required. Correa-Martínez et al*.* describe an antimicrobial susceptibility test on a MALDI plate: bacteria are incubated in microdroplets containing a range of concentrations of an antibiotic, then expression of resistance markers is measured via MALDI^9,10^, but at least 3 h incubation and several delicate pipetting manipulations are required.

To achieve the speed and limited hands-on time necessary for clinical use, we developed a new extraction method—FLAT (“fast lipid analysis technique”), that requires less than an hour of elapsed time and is highly parallelizable and amenable to automation. By directly extracting lipids on the heated surface of a stainless steel MALDI plate, FLAT minimizes handling steps and thus demands significantly less hands-on time than previous lipid analysis methods. Furthermore, FLAT can identify bacteria and fungi, while simultaneously detecting antimicrobial resistance signals that are not reported by current clinical MALDI ID systems, thus demonstrating the significant potential of FLAT in a clinical diagnostic laboratory setting.

## Materials and methods

### Microbial strains and culture

We applied the FLAT method to 149 replicates of 35 bacterial strains as follows: 20 unique clinical and laboratory adapted isolates of Gram-negative bacteria (*E. coli, K. pneumoniae, A. baumannii*, *P. aeruginosa, M. morganii,* and *S. marcescens)* including seven strains transformed with the *mcr-1* plasmid^26,30^, and 15 unique clinical and laboratory adapted isolates of Gram-positive bacteria (*S. aureus, B. cereus, and B. mycoides).* In addition, we applied the FLAT method to four replicates of two laboratory adapted isolates of *Candida auris*. We compared FLAT results to results with lipid microextraction^31^ on 157 replicates of the same and similar strains of the same bacterial and fungal species. A complete list of strains and replicates is provided in Supplementary Table S1 online. Bacterial samples were cultured overnight at 37 °C on Difco LB agar plates (BD, Franklin Lakes NJ). Liquid bacterial samples were cultured overnight at 37 °C in Difco LB broth (BD, Franklin Lakes NJ) shaking at 180 RPM. *C. auris* samples were cultured overnight at 37 °C on Tryptic Soy Agar (MilliporeSigma, Burlington MA).

### FLAT extraction of microbial lipids

Microbial colony smears or liquid samples were applied to a target location on a stainless steel MALDI plate. The target plate was incubated in a humidified chamber for 30 min at 110 °C. The MALDI plate was washed with deionized water from a squeeze bottle, allowed to air dry, then 1 μL of norharmane matrix solution was applied (10 mg/mL in 12:6:1, v/v/v chloroform/methanol/water) to each target location. Following the method of Leung et al*.*^16^, spectra were acquired from target locations in negative ion mode using a Microflex LRF MALDI-TOF MS (Bruker, Billerica MA) in reflectron mode with a limited mass range of 1000–2400 *m/z.* Typically, 300 laser shots were summed to acquire each mass spectrum.

### Microextraction of microbial lipids

The lipid microextraction method used by Leung et al*.* was originally described by El Hamidi et al*.*^16,27,31^. Briefly, bacterial pellets were treated with an isobutyric acid/NaOH mixture, incubated 30–45 min at 100 °C, then centrifuged at 2000×*g* for 15 min to remove cell debris. Supernatants were transferred to clean tubes, diluted 1:1 with distilled water, and lyophilized overnight. The resulting dry pellets were washed twice in methanol, resuspended in 12:6:1, v/v/v chloroform/methanol/water, then 1 µL was dispensed onto a stainless steel MALDI plate. Matrix application and spectra acquisition conditions were otherwise identical to those used in the FLAT samples.

### Computational analysis of mass spectra

Spectra were exported from the mass spectrometer as mzXML files and read with a Python program (source available below) using the mzXML reader in Pyteomics 4.3.2^32^. Spectra were resampled onto a regular grid and background corrected with SNIP (Statistics-sensitive, Non-linear Iterative Peak-clipping)^33^. The similarity between two spectra is measured by cosine similarity: the cosine of the angle formed by the spectra treated as normalized vectors. In this way, a similarity between each spectrum and every other spectrum of interest was computed as a scalar number.

To measure the similarity between one group of spectra and another group, such as between spectra for one species and another species, the cosine similarities from each spectrum in one group to each spectrum in the other group were averaged, then the averages were averaged (if a spectrum appears in both groups, its self-similarity was omitted from the averages).

To evaluate two sets of spectra relative to species-specific signals, each set was sub-grouped by species, then the similarity was measured between each subgroup of one set and every subgroup of the other set. The result is a matrix of similarities, as is often used to compare the similarity of individual spectra, but here shows the average similarity of multiple spectra from the same species. Results are presented as bubblemaps in Fig. 4, with larger bubbles indicating greater similarity, and similarity values labeled numerically on a scale of 0.0–1.0 where similarity exceeds 0.4.

## Results

### The FLAT method for extracting microbial signature lipids

**Figure 1:**
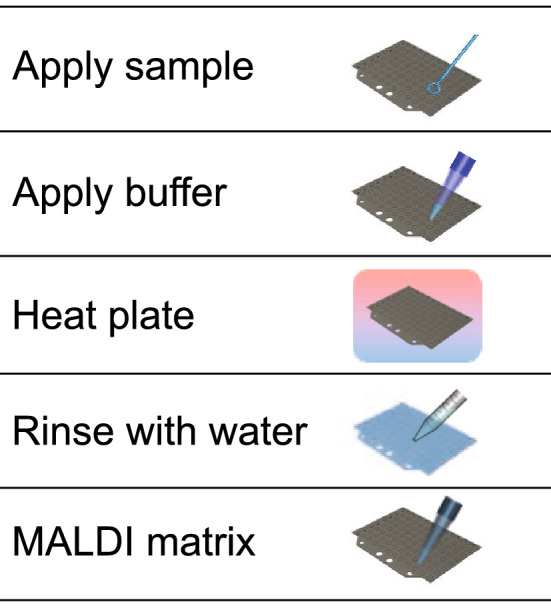
FLAT extraction workflow. A sample is applied to a stainless steel MALDI target plate, followed by FLAT extraction buffer (for Gram-negative species; 0.2 M anhydrous citric acid, 0.1 M trisodium citrate dihydrate) or no buffer (for Gram-positive and fungal species). The entire plate is incubated in a humidified chamber at 110 °C for 30 min and then rinsed thoroughly with water. MALDI matrix is applied to sample spots and lipid spectra are collected using MALDI-TOF MS.

FLAT (Fig. 1) resembles current MALDI protein ID systems^34–36^ in that a microbial colony smear or liquid sample is directly applied to a target location on a MALDI plate. As described in “Materials and methods”, we applied FLAT to 149 replicates of 35 bacterial strains and four replicates of two unique *C. auris* strains. Total processing time, from the start of sample handling to the end of mass spectra acquisition, was less than 1 h in all cases. We extracted 157 replicates of the same and similar strains via lipid microextraction, as described in “Materials and methods”. MALDI spectra from these extractions were then analyzed and compared as follows.

### Buffered and unbuffered FLAT extraction

**Figure 2:**
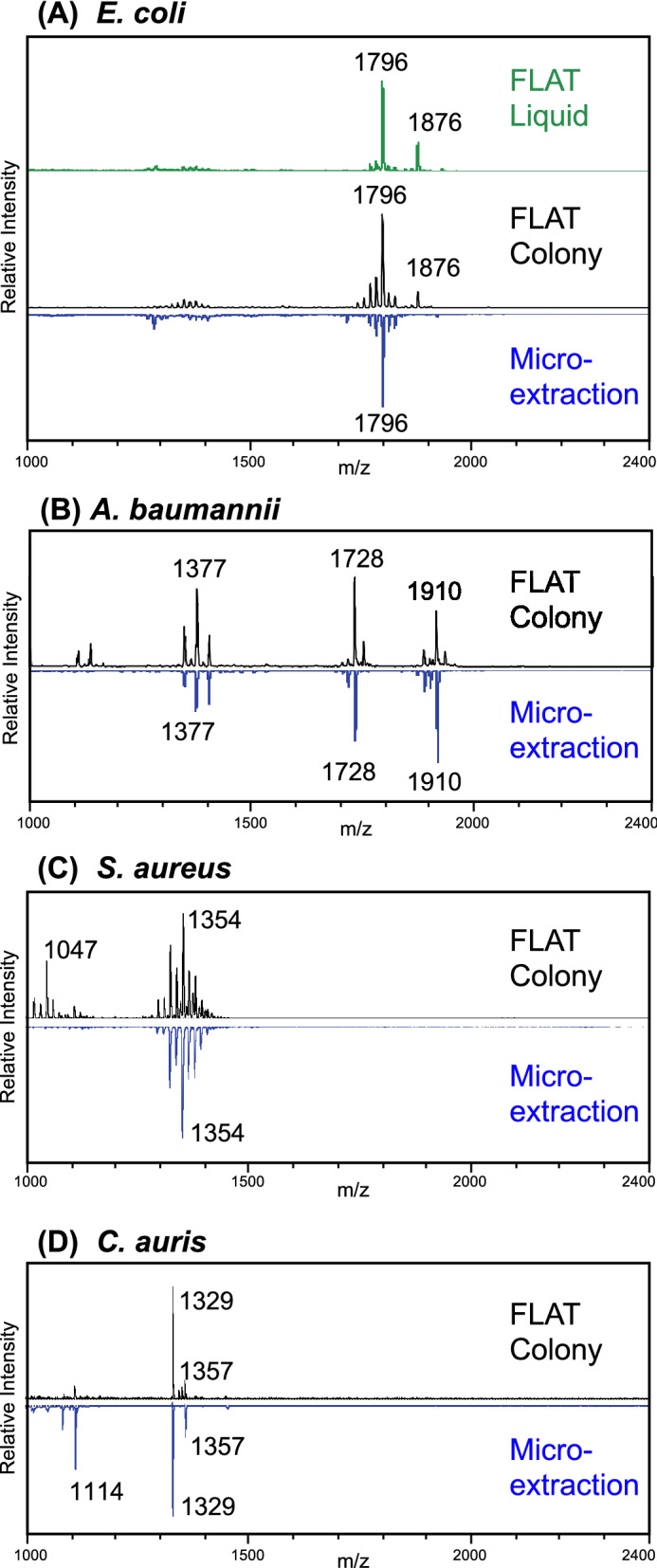
Representative FLAT spectra compared to microextractions spectra of the same strain. (**A**) *Escherichia coli* ATCC 25922 colony smear and liquid culture via FLAT is compared to microextraction. The characteristic 1796 *m/z* lipid A ion^28^ is observed in all spectra. Additionally, an ion at 1876 *m/z* is observed with FLAT, but not with microextraction. This is plausibly a lipid A ion, and may have been previously observed in *E. coli*^28^, but is not typically extracted via microextraction. Colony smears via FLAT are compared to microextraction in (**B**) through (**D**), with the same base peak shown for both methods for each species. (**B**) *Acinetobacter baumannii* SM1536. Previously reported ions^16^ are observed. (**C**) *Staphylococcus aureus* NRS484. The pattern of ions from 1300 to 1400 *m/z* is highly similar in both methods. An ion at 1047 *m/z* in FLAT may be present at very low intensity in microextraction. (**D**) *Candida auris* AR0384. Two ions at 1329 and 1357 *m/z* are present in both spectra. An ion at 1114 *m/z* in microextraction may be present at low intensity in FLAT.

Acidic conditions facilitate extraction of lipid A molecular species^16,28,31^, which are important for identifying Gram-negative bacteria^16^. However, acidic conditions may interfere with lipid extraction for some Gram-positive and fungal species. We therefore performed FLAT extraction both with and without a buffer on all samples. For each sample, two target spots were applied and treated as follows: on one target spot, 1 µL of buffer (0.2 M citric acid, 0.1 M trisodium citrate) was dispensed before extraction; the buffer was not applied to the second spot. Characteristic lipid A ions of Gram-negative bacteria were only observed in spectra from buffered spots. Characteristic lipid ions of Gram-positive bacteria and fungi were observed in spectra from both buffered and unbuffered spots. However, for Gram-positive bacteria and fungi, unbuffered spots consistently produced higher spectral quality than buffered spots. Consequently, in the following studies, spectra from buffered spots were used for Gram-negative bacteria (Figs. 2A,B and 4), while spectra from unbuffered spots were used for Gram-positive bacteria and fungi (Fig. 2C,D).

### Comparison of FLAT to lipid microextraction

We compared MALDI spectra produced by FLAT extraction to the spectra produced by lipid microextraction to determine if the methods extract similar lipid profiles. In lipid microextraction, methanol washing removes phospholipids in the relevant mass range, suppressing their signal. FLAT does not require depletion of phospholipids in the same way, so some differences may be expected between FLAT and lipid microextraction. Figure 2 shows representative FLAT and lipid microextraction method spectra, demonstrating that the two methods produce highly similar spectra with the same characteristic ions and giving confidence that FLAT can be applied to microbial and resistance ID as described*.* Supplementary Figure S1 compares FLAT and microextraction of *E. coli* over the mass range 800–2200 *m/z,* showing that FLAT extracts lipids below 1000 *m/z*. Supplementary Figure S2 shows a FLAT mass spectrum of *E. coli* from 600 to 800 *m/z*. However, phospholipids below 1000 m/z generally do not differentiate bacteria by species^16^.

### Comparison of colony smears to liquid culture

We compared FLAT extraction of a liquid sample to FLAT extraction of a colony smear of the same strain. From overnight liquid culture of *E. coli* ATCC 25922, 1 µL was applied to a MALDI target spot and allowed to dry. A colony smear of the same *E. coli* strain was applied to a second target spot. The FLAT method with buffer was performed on the MALDI plate and spectra collected. Lipid microextraction was performed on the same *E. coli* strain. Figure 2A shows the resulting MALDI spectra, all of which have the characteristic *E. coli* lipid A ion at 1796 *m/z* as their principal ion.

### Antimicrobial resistance detection

**Figure 3:**
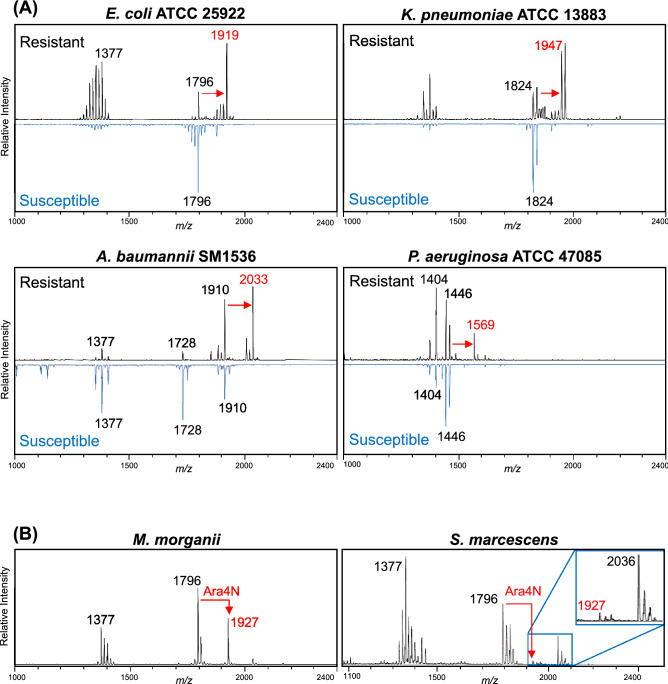
Detection of antibiotic resistance. (**A**) FLAT was used on isolates expressing the gene *mcr-1* from a plasmid leading to colistin resistance via phosphoethanolamine (PEtN) addition to lipid A (observed as Δ*m/z* 123, shown in red). In resistant spectra, the intensity of the unmodified lipid A base peak is less than that of the modified ion, increasing the relative intensity of other ions in the spectra, especially ions between 1300 and 1400 m*/z* in *E. coli* and *K. pneumonia*. This effect is not clear in *A. baumannii* and *P. aeruginosa,* because in each case the intensity of the most prominent unmodified lipid A ion is similar to the intensity of the modified ion. (**B**) Intrinsically colistin-resistant bacteria *Morganella morganii* and *Serratia marcescens* were analyzed via FLAT for resistance-associated peaks. Lipid A modification with l-amino-4-arabinose (Ara4N) was observed (as Δ*m/z* 131) in *M. morganii* spectra. Lipid A modification with Ara4N was observed (as Δ*m/z* 131) in both species.

Lipid spectra are uniquely suited to identifying colistin resistance-associated ions, as previously described^18,19,21–23,25,27–30^. We evaluated FLAT as a rapid antimicrobial resistance assay, using bacterial strains that are either intrinsically colistin-resistant or have colistin resistance conferred through acquisition of a plasmid expressing the *mcr-1* gene^26,30^. The *mcr-1* plasmid encodes a phosphoethanolamine (PEtN) transferase and is responsible for the addition of a PEtN group to the terminal phosphate moieties of lipid A, thereby masking their negative charge and reducing membrane interactions through electrostatic binding to cationic antibiotics, such as colistin (polymyxin E). The addition of PEtN can be tracked with mass spectrometry via an increase in mass of the signature ion(s) present in the unmodified organism (Δ*m/z* 123)^27^. To confirm the broad applicability of FLAT, we analyzed seven *mcr-1*-expressing strains from four different species (*E. coli, K. pneumoniae, A. baumannii*, and *P. aeruginosa*) and detected PEtN-modified lipid A in 100% of spectra (Fig. 3A, labeled in red). We also analyzed two bacterial species intrinsically resistant to colistin: *M. morganii* and *S. marcescens.* Lipid A from these bacterial backgrounds was modified with amino-containing sugars, including Ara4N, thereby conferring colistin resistance on these species in the absence of *mcr-1* (Fig. 3B).

### Computational comparison of FLAT and lipid microextraction

To compare FLAT and lipid microextraction semi-quantitatively, we evaluated spectra from 149 replicates of 35 strains of three Gram-positive and six Gram-negative bacterial species, including seven *mcr-1* expressing strains and five strains intrinsically resistant to colistin. We compared these FLAT spectra to 148 unique lipid microextraction spectra from the same nine species. Generally, the same strains were used to generate both FLAT and lipid microextraction spectra, although additional strains were added for lipid microextraction to make a more rigorous comparison. Evaluated strains are summarized above and in Supplementary Tables S1 and S2 online.

**Figure 4:**
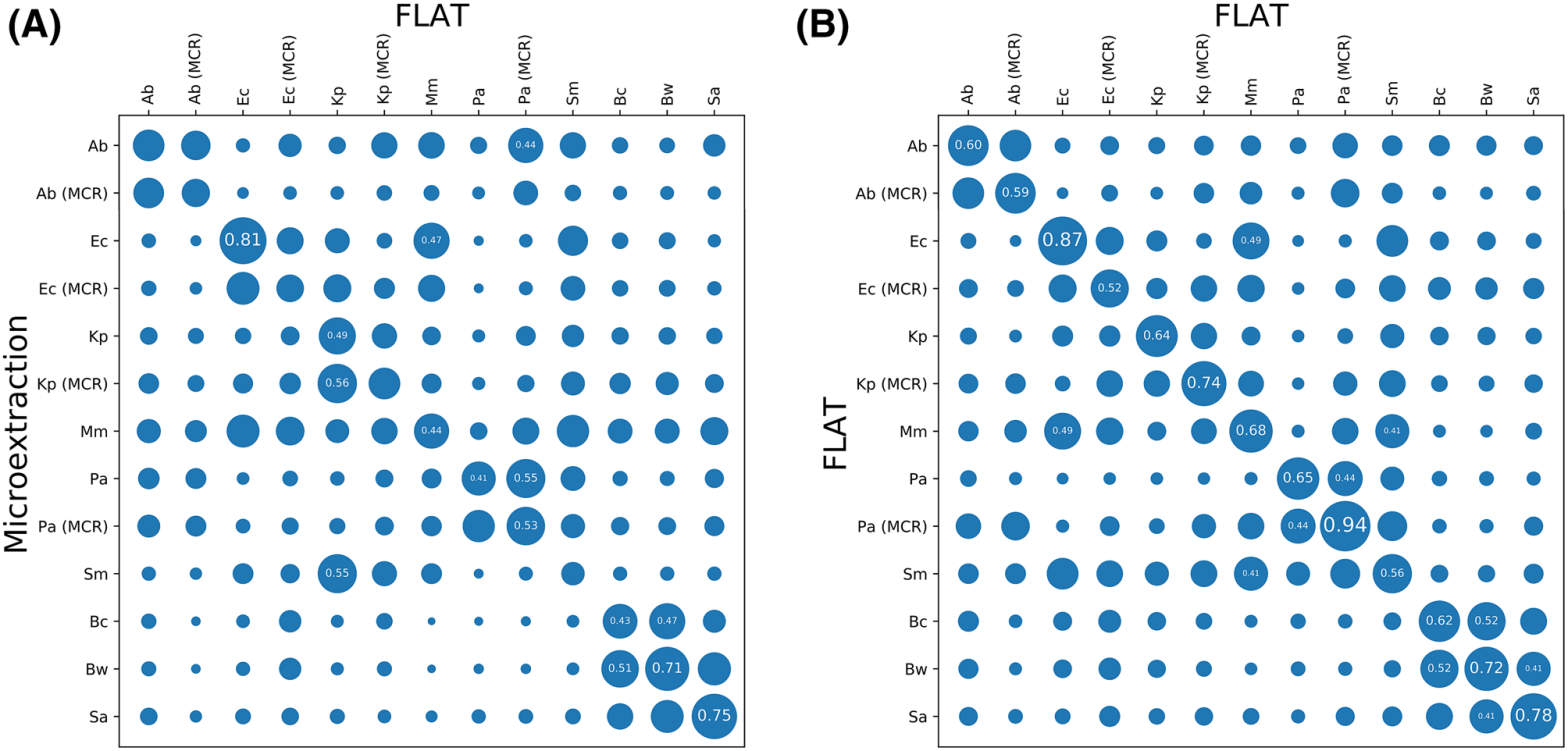
FLAT spectra are distinctive by bacterial species and similar to equivalent Microextraction spectra. (**A**) A heatmap demonstrating that FLAT produces spectra consistent with microextraction. (**B**) A heatmap demonstrating FLAT species and *mcr-1* status differentiation. In both cases, similarity between two groups of spectra was computed via the mean cosine similarity across all pairs of spectra, and is visualized as a circle with a proportionally scaled diameter. A total of 149 FLAT spectra and 148 microextraction spectra were used in this comparison, from strains and samples as described in Supplementary Tables S1 and S2 online. CR: *mcr-1* transformed; Ab: *Acinetobacter baumannii;* Ec: *Escherichia coli;* Kp: *Klebsiella pneumoniae* Pa: *Pseudomonas aeruginosa;* Mm: *Morganella morganii;* Sm: *Serratia marcescens;* Bc: *Bacillus cereus;* Bw: *Bacillus mycoides* (previously known as *Bacillus weihenstephanensi*s^37^); Sa: *Staphylococcus aureus.*

FLAT and lipid microextraction spectra were compared by species and colistin resistance status using the averaged cosine similarity method described in “Materials and methods”, producing the two-dimensional bubble map in Fig. 4A. Larger circles represent more similar spectra. The prominent diagonal indicates that FLAT and lipid microextraction spectra are overall similar, with differences in the relative intensity of extracted lipids.

### Differentiation of species and colistin resistance

We evaluated the ability of FLAT to differentiate species and colistin resistance status by evaluating the 149 FLAT spectra of three Gram-positive and six Gram-negative bacterial species described above. In this case, the FLAT spectra were compared to themselves, to specifically measure the degree to which FLAT produces differential signals by species and colistin resistance. Results are shown in Fig. 4B, where a much more prominent diagonal than in Fig. 4A is observed. In each case, the value on the diagonal is the most similar (largest) value in its column and row, indicating that FLAT produced distinct spectra for species, for colistin resistance status, and for each unique combination of species and colistin resistance status.

## Discussion

### FLAT rapidly extracts microbial lipid barcodes

We have shown by inspection and via average cosine similarity that, for a variety of microbial species, FLAT and lipid microextractions produce similar spectra at the species level and also that FLAT produces reliably distinct spectra for each species. FLAT thus shows obvious potential for automated species identification and antimicrobial resistance detection. In future work, we intend to demonstrate such automated identification, although not using cosine similarity as a starting point. FLAT readily detects modifications to microbial lipids, allowing detection of some but not all types of antimicrobial resistance. As with the 123 *m/z* difference due to phosphoethanolamine addition, specific lipid modifications conferring resistance can be detected as mass differences, even in species in which that specific resistance has not previously been observed.

Like lipid microextraction spectra, FLAT spectra provide a barcode that uniquely identifies microbial species. Unlike lipid microextraction and proposed alternatives, FLAT extraction can be accomplished in less than an hour without centrifugation. FLAT is thus a practical clinical method and amenable to straightforward automation, unlike lipid microextraction and alternatives.

When testing clinical samples, Gram status may be unknown, so a FLAT-based clinical assay can use two MALDI target spots per sample: one target spot with buffer is for Gram-negative bacteria while another spot without buffer is for Gram-positive bacteria and fungi. Samples would then be applied to both target spots. Normally, only one spot produces a high-quality spectrum and that spot would be used for identification. If both spots produce high-quality spectra, then either or both can be used for identification, requiring minimal additional processing time.

### FLAT rapidly detects antimicrobial resistance

Detection of antimicrobial resistance is critical for providing effective patient treatment regimes. MALDI-based methods for antibiotic resistance have been proposed^4–7^, but to date have not resulted in a clinical diagnostic test. However, lipid spectra are uniquely suited to rapidly identify resistance-associated ions, as previously described, particularly in Gram-negative bacteria^25,27,28^. We evaluated FLAT as a rapid antimicrobial resistance assay, using bacterial strains that are either intrinsically colistin-resistant or have colistin resistance conferred by the *mcr-1* plasmid. FLAT reliably detected both intrinsic colistin resistance and resistance conferred by *mcr-1.* In total, we demonstrate that flat extraction is capable of detecting antibiotic-resistance-associated structures in Gram-negative bacteria, thereby providing species-level identification and antibiotic susceptibility information in a single assay.

### FLAT demonstrates potential as a clinical assay

Similar to lipid microextraction, FLAT extracts microbial lipid barcodes. Further, FLAT barcode spectra can be used in place of lipid microextraction spectra to identify bacteria and fungi and detect colistin resistance, using the BACLIB method. Ultimately, FLAT has important advantages to lipid microextraction and other alternatives for the BACLIB method, in that it takes less than an hour, uses clinic-friendly reagents, has low hands-on time, and does not require centrifugation. Therefore, FLAT is a potential clinical approach to the BACLIB microbial ID and resistance technique of Leung et al*.* FLAT thus offers important advantages to current clinical assays, in a clinic-friendly format.

## Supplementary Information

Supplementary Information.
