# Supplementary material for: Rapid microbial identification and colistin resistance detection via MALDI-TOF MS using a novel on-target extraction of membrane lipids
Source: Sci Rep. 2020 Dec 9;10:21536. doi: 10.1038/s41598-020-78401-3 (PMC7725828; doi:10.1038/s41598-020-78401-3)
Supplement: Supplementary file 1 — Supplementary Information. [file 41598_2020_78401_MOESM1_ESM.pdf]

Rapid microbial identification and colistin resistance detection via MALDI-TOF MS using a novel on-target extraction of membrane lipids

SUPPLEMENTARY INFORMATION

AUTHORS

Matthew Sorensen[1,#,*], Courtney E. Chandler[1,2,#,^], Francesca M. Gardner[1,2], Salma Ramadan[1], Prasanna D. Khot[3], Lisa M. Leung[4,+], Christine E. Farrance[3] , David R. Goodlett[2,5], Robert K. Ernst[2,*], Erik Nilsson[1]

# These authors contributed equally to this work

+ Current address: U.S. Food and Drug Administration, Silver Spring, MD 20993

^ Current address: Johns Hopkins University, Baltimore, MD 21225

* Correspondence to matthew@pataigin.com and Rkernst@umaryland.edu

AUTHOR AFFILIATIONS

1 – Pataigin, LLC, Seattle, WA

2 – University of Maryland, Baltimore, Baltimore, MD, 21201

3 – Charles River Laboratories, Inc., Newark, DE, 19711

4 – Maryland Department of Health and Mental Hygiene, Baltimore, MD, 21201

5 - University of Gdansk, International Centre for Cancer Vaccine Science, Gdansk, Poland, EU

Organisms	Strain (note 1)	Source	FLAT Replicates	Micro Replicates
**Gram-negative**				
Acinetobacter baumannii	ATCC 17978	ATCC	6	3
	ATCC 17978 (pMQ124WH1266-*mcr-1*)	ATCC	2	2
	SM1536	Y. Doi	2	5
	SM1536 (pMQ124WH1266-*mcr-1*)	Y. Doi	2	1
Escherichia coli (note 2)	ATCC 25922	ATCC	19	2
	ATCC 25922 (pMQ124-*mcr-1*)	ATCC	2	2
	YD626	Y. Doi	2	2
	YD626 (pMQ124-*mcr-1*)	Y. Doi	2	2
	K12	R. Ernst	2	2
Klebsiella pneumoniae	ATCC 13883^T	ATCC	6	0
	ATCC 13883 (pBCSK-*mcr-1*)	ATCC	2	1
	4081916	R. Ernst	2	0
	4081916 (pMQ124-*mcr-1*)	R. Ernst	2	0
	TBE812	R. Ernst	0	2
	TBE818	R. Ernst	0	10
Pseudomonas aeruginosa	ATCC 47085	ATCC	7	4
	ATCC 47085 (pMQ124-*mcr-1*)	ATCC	2	3
	TRPA087	Y. Doi	2	11
	TRPA179 (pMQ124-*mcr-1*)	Y. Doi	0	2
Morganella morganii	YDC562	Y. Doi	4	6
	YDC700	Y. Doi	4	6
	YDC721	Y. Doi	0	6
	YDC723	Y. Doi	0	5
Serratia marcescens	YDC507	Y. Doi	4	3
	YDC563	Y. Doi	4	4
	YDC583	Y. Doi	4	3
	YDC591	Y. Doi	0	3
	YDC609	Y. Doi	0	4
	YDC629	Y. Doi	0	3
	YDC639	Y. Doi	0	3
	YDC647	Y. Doi	0	3
	YDC719	Y. Doi	0	3
**Gram-positive**				
Bacillus cereus	ATCC 14579^T	C. Farrance	17	9
Bacillus mycoides (note 3)	LMG 18989^T	C. Farrance	16	9
Staphylococcus aureus	ATCC 29213	ATCC	2	2
	NRS1	M. Shirtliff	2	2
	NRS100	M. Shirtliff	3	2
	NRS123	M. Shirtliff	3	2
	NRS382	M. Shirtliff	3	2
	NRS383	M. Shirtliff	4	2
	NRS384	M. Shirtliff	2	2
	NRS385	M. Shirtliff	2	2
	NRS386	M. Shirtliff	4	2
	NRS387	M. Shirtliff	2	2
	NRS482	M. Shirtliff	3	2
	NRS483	M. Shirtliff	4	2
	NRS484	M. Shirtliff	2	2
**Fungi**				
Candida auris	AR0384	L. Leung	2	6
	AR0385	L. Leung	2	3

Supplementary Table S1: Organisms and Strains used, including the number of replicates used with each of the FLAT and lipid microextraction methods.

Organisms	Strain	PCR for mcr-1 (note 4)	FLAT Replicates	Micro Replicates
**Gram-negative**				
Acinetobacter baumannii	ATCC 17978	-	6	3
	ATCC 17978 (pMQ124WH1266-*mcr-1*)	+	2	2
	SM1536	-	2	5
	SM1536 (pMQ124WH1266-*mcr-1*)	+	2	1
Escherichia coli	ATCC 25922	-	19	2
	ATCC 25922 (pMQ124-*mcr-1*)	+	2	2
	YD626	-	2	2
	YD626 (pMQ124-*mcr-1*)	+	2	2
Klebsiella pneumoniae	ATCC 13883^T	-	6	0
	ATCC 13883 (pBCSK-*mcr-1*)	+	2	1
	4081916	-	2	0
	4081916 (pMQ124-*mcr-1*)	+	2	0
	TBE812	-	0	2
	TBE818	-	0	10
Pseudomonas aeruginosa	ATCC 47085	-	7	4
	ATCC 47085 (pMQ124-*mcr-1*)	+	2	3
	TRPA087	-	2	11
	TRPA179 (pMQ124-*mcr-1*)	+	0	2
Morganella morganii	YDC562	n/a	4	6
	YDC700	n/a	4	6
	YDC721	n/a	0	6
	YDC723	n/a	0	5
Serratia marcescens	YDC507	n/a	4	3
	YDC563	n/a	4	4
	YDC583	n/a	4	3
	YDC591	n/a	0	3
	YDC609	n/a	0	4
	YDC629	n/a	0	3
	YDC639	n/a	0	3
	YDC647	n/a	0	3
	YDC719	n/a	0	3
**Gram-positive**				
Bacillus cereus	ATCC 14579^T	n/a	17	9
Bacillus mycoides (note 3)	LMG 18989^T	n/a	16	9
Staphylococcus aureus	ATCC 29213	n/a	2	2
	NRS1	n/a	2	2
	NRS100	n/a	3	2
	NRS123	n/a	3	2
	NRS382	n/a	3	2
	NRS383	n/a	4	2
	NRS384	n/a	2	2
	NRS385	n/a	2	2
	NRS386	n/a	4	2
	NRS387	n/a	2	2
	NRS482	n/a	3	2
	NRS483	n/a	4	2
	NRS484	n/a	2	2
**Total Replicates used in heatmaps**			**149**	**148**

Supplementary Table S2: Organisms and Strains used in quantitative comparison. Shown are the replicates from **Supplementary Table S1** used to prepare the heatmaps in **Figure 4.**

Notes for Supplementary Tables S1 and S2

For description of the methods used for the “FLAT” and “Micro” (lipid microextraction) replicates, see main text.

(note 1) Strains that note a cloning vector construct in parenthesis were transformed with the *mcr-1* plasmid in the Ernst lab. The source for these strains indicates the source of the strain prior to transformation.

(note 2) Of the 19 *E. coli* ATCC 25922 isolates extracted via FLAT, 16 were extracted from liquid cultures and one was extracted from a colony smear. All other isolates used for the computational comparison were extracted from colony smears. As shown in Figure 2A, we have observed that FLAT produces highly similar results from colony smears and liquid culture. The *E. coli* K12 isolate was used only for **Supplementary Figure S1**.

(note 3) *Bacillus mycoides* previously known as *Bacillus weihenstephanensis.* See Liu, Y., Lai, Q. & Shao, Z. Genome analysis-based reclassification of *Bacillus weihenstephanensis* as a later heterotypic synonym of *Bacillus mycoides. Int J Syst Evol Microbiol.* **68,** 106-112 (2018). doi:10.1099/ijsem.0.002466

(note 4) The *mcr-1* plasmid does not transform Gram-positive bacteria. Theoretically, the *mcr-1* plasmid could transform Gram-negative species *M. morganii* and *S. marcescens,* but these species are innately resistant to polymyxins. Consequently, none of the *M. morganii, S. marcescens,* or Gram-positive strains were evaluated for the *mcr-1* gene by PCR.

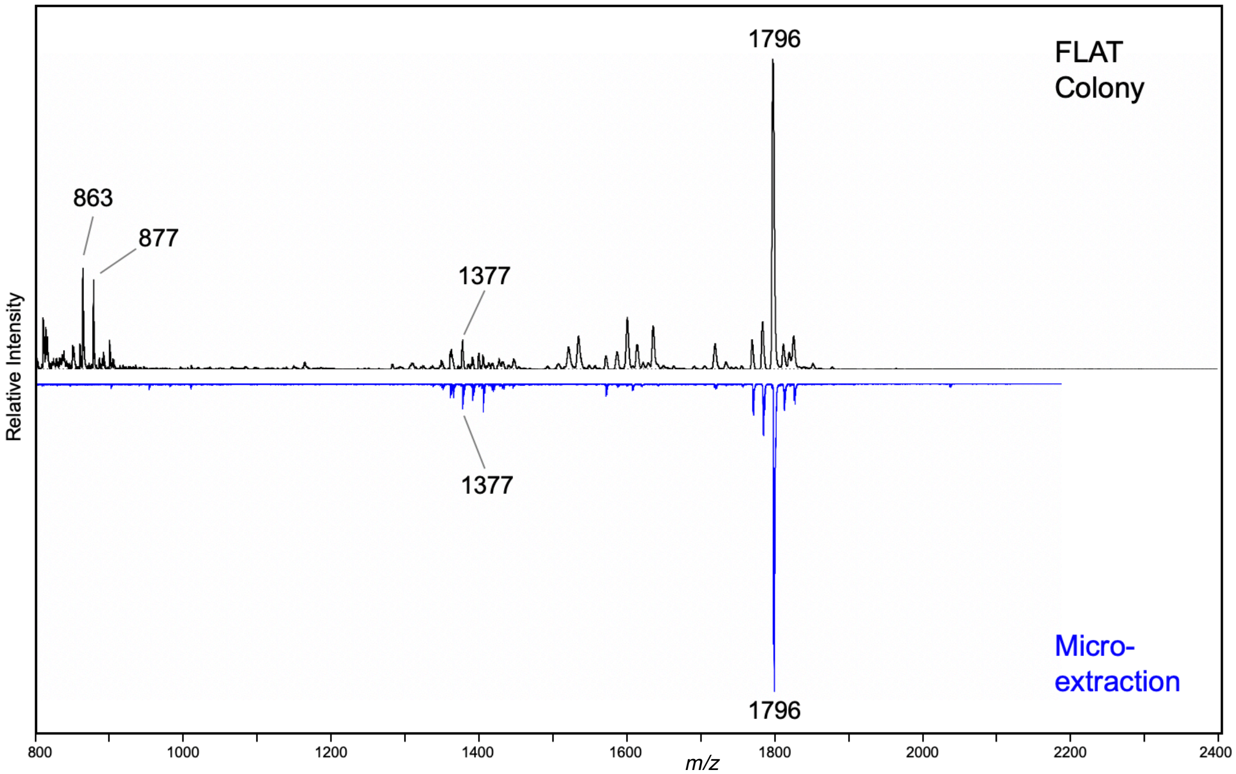

Supplementary Figure S1: Spectra comparing FLAT to microextraction. A spectrum from an *E. coli* K12 colony smear prepared via FLAT is compared to a spectrum of the same strain prepared via microextraction. In both spectra, the base peak is the characteristic 1796 *m/z* lipid A ion. Prominent ions in the microextraction spectrum are also present in the FLAT spectrum. Additional ions are observed in the FLAT spectrum that are not observed in the microextraction spectrum.

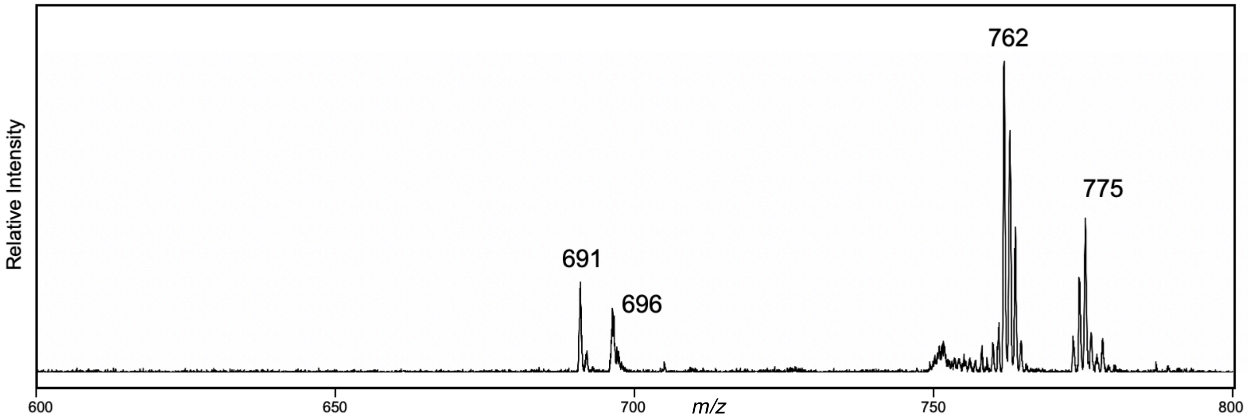

Supplementary Figure S2: Sub 800 *m/z* FLAT spectrum. Shown is a spectrum from 600 to 800 *m/z* of an *E. coli* ATCC 25922 colony smear prepared via FLAT. Prominent ions corresponding to phospholipids are indicated.
